# The circRNA circADAMTS6 promotes progression of ESCC and correlates with prognosis

**DOI:** 10.1038/s41598-022-17450-2

**Published:** 2022-08-12

**Authors:** Jing Bu, Lina Gu, Xin Liu, Xixi Nan, Xiangmei Zhang, Lingjiao Meng, Yang Zheng, Fei Liu, Jiali Li, Ziyi Li, Meixiang Sang, Baoen Shan

**Affiliations:** 1 Department of Research Center, The Fourth Hospital of Hebei Medical University, Shijiazhuang, Hebei People’s Republic of China; 2 Tumor Research Institute, The Fourth Hospital of Hebei Medical University, 050017 Shijiazhuang, Hebei People’s Republic of China

**Keywords:** Cancer, Medical research, Oncology, Risk factors

## Abstract

Circular RNAs (circRNAs) are a type of noncoding RNA, which play a vital role in the occurrence and development of esophageal squamous cell carcinoma (ESCC). While the role of novel circADAMTS6 in ESCC remains unknown. We assessed circADAMTS6 expression in ESCC tissues and cells, and the relationship between circADAMTS6 expression and overall survival of ESCC patients. Functional experiments in vitro and xenograft in vivo assay were applied to explore the functions and mechanisms of circADAMTS6 in ESCC. Results found that up-regulation of circADAMTS6 was associated with poor overall survival and may acted as an independent risk factor for ESCC prognosis. Knockdown of circADAMTS6 significantly inhibited the proliferation, migration and invasion of ESCC cells and growth of xenograft tumors in vivo. Induced AGR2 expression was able to rescue the loss of function induced by si-circADAMTS6 in KYSE150 cell. CircADAMTS6 may acts as oncogene by activating AGR2 and the Hippo signaling pathway coactivator YAP in ESCC.

## Introduction

Esophageal cancer is the sixth dominant cause of cancer-related mortality worldwide that threatens human health seriously^1^. It has a high incidence in China, especially in Shanxi and Hebei Province. As the main pathological type of esophageal cancer in China, ESCC accounts for more than 90% of the total number of esophageal cancers^2–4^. Despite rapid advances in treatments, including neoadjuvant chemotherapy or immunity therapy, the prognosis of ESCC patents remains poor, with 5-year overall survival (OS) rate is less than 20%^5^. Consequently, our immediate concern is to investigate the pathogenesis of ESCC and to seek early screening indicators.

As a group of endogenous noncoding RNAs, circRNAs have stable covalent closed loop structure^6,7^, which makes them resistant to RNA exonuclease compared to linear RNA. In addition, the tissue expression specificity of circRNAs endows them stably exist in saliva, plasma and other peripheral tissue, making them potential prognostic markers^8–10^. Growing evidence suggests that circRNAs are abnormally expressed in various diseases including ESCC and may act as tumor suppressor genes or oncogenes during the occurrence and development of cancer^11,12^. Our previous studies showed that ciRS-7 was over-expressed in ESCC tissue and accelerated the proliferation, migration and invasion ability of ESCC cells by regulating the miR-7/KLF4 axis to activate the NF-κB p65 signaling pathway^13^. Cao et al. found that the up-regulation of circRNA-100876 promoted ESCC cell invasion, migration and epithelial mesenchymal transition (EMT), and associated with poor prognosis^14^. For instance, circUBAP2 plays a role of oncogene by regulating mir-422a/rab10 axis and may be a predictive marker for the prognosis of ESCC^15^. Above discovers reveal the vital role of circRNAs in ESCC. However, many valuable circRNAs related to ESCC need to be further explored and identified.

Anterior Gradient Homolog 2 (AGR2) is a member of protein disulfide isomerase (PDI) family, which is overexpressed in ESCC, lung cancer, breast cancer and other cancer^16^. Studies demonstrated that AGR2 promotes tumor growth by inducing dephosphorylation of Yes-associated protein (YAP) in lung adenocarcinoma^17^. Besides, AGR2 overexpression promoted cell proliferation and migration and inhibited TNF-induced intestinal epithelial barrier damage by activating YAP^18^. However, little is known about the relationship of AGR2 and circRNAs in ESCC.

In our research, hsa_circ_0072688 (also called circADAMTS6, originated from the ADAMTS6 gene) was discovered and dramatically expressed in ESCC tissues and cells. qRT-PCR results showed that knockdown of circADAMTS6 significantly reduced the proliferation, migration and invasion of KYSE150 and KYSE30 cells. Base on recent investigations indicating the function of AGR2 to cancer progression, we aimed to confirm regulatory effect of circADAMTS6 on AGR2 expression. Mechanistically, circADAMTS6 positively regulates the expression of AGR2 to accelerate the proliferation and invasion of KYSE150 cell by activating the expression level of the Hippo signaling pathway co-activator YAP. In conclusion, our study demonstrated that circADAMTS6 may play a vital role as an oncogene and serve as a tumor marker to promote early diagnosis and treatment of ESCC.

## Results

### The features of circADAMTS6

**Figure 1:**
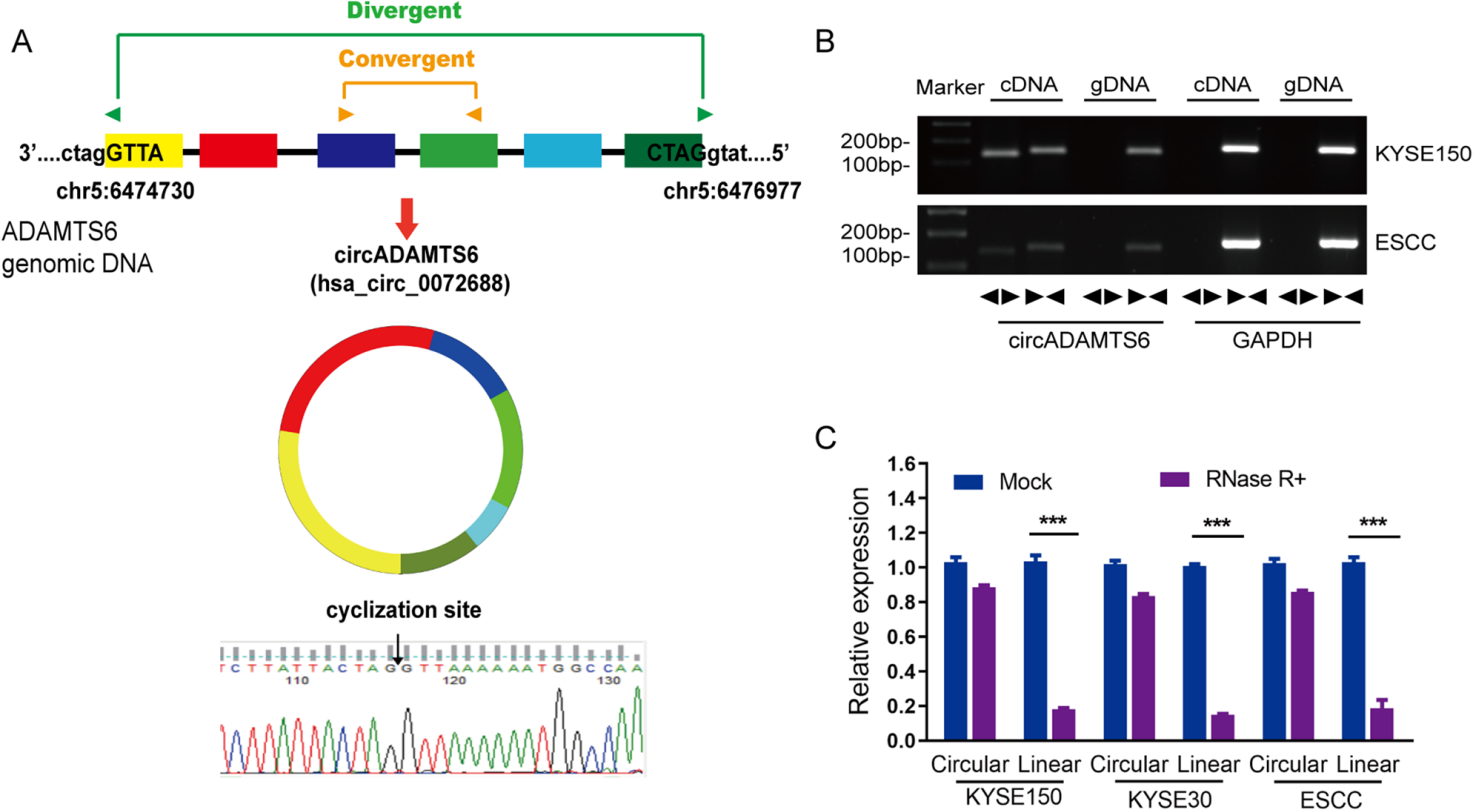
The characteristics of circADAMTS6 in ESCC cells. (**A**) circADAMTS6 is formed by reverse splicing of exons 2–7 in the ADAMTS6 gene. (**B**) The existence of circADAMTS6 in ESCC cell and tissue was confirmed. CircADAMTS6 was only amplified by divergent primers in cDNA but not in gDNA. GAPDH was acted as a linear RNA control. Divergent primers or convergent primers were indicated by the opposite or the same directions of the arrowhead. (**C**) RT-PCR assessed the expression levels of circADAMTS6 and linear mRNA upon RNase R treatment in ESCC cells and tissues.

The circADAMTS6 located at chr5:6,474,730–6,476,977 and originated from exon 2–7 of ADAMTS6 gene by back-splicing (Fig. 1A). The back-splice junction site of circADAMTS6 was amplified using the divergent primers and verified by Sanger sequencing. In addition, the divergent primers and convergent primers were designed to amplify the circular and linear transcripts of ADAMTS6 in cDNA and gDNA, respectively. GAPDH was acted as a linear RNA control. As expected in the PCR results, the circular form was only amplified by divergent primers in cDNA but not in gDNA, while the linear form was amplified by convergent primers in both cDNA and gDNA (Fig. 1B). Furthermore, we employed a highly processive 3’ to 5’ exoribonuclease (RNase R enzyme)^19^ to explore the characteristics of circADAMTS6 in ESCC cell and tissue. The results showed that the circADAMTS6 was resistant to RNase R treatment, which was different from the linear control gene GAPDH (Fig. 1C), indicating that the ADAMTS6 gene has a circular RNA form that is independent of ADAMTS6 mRNA.

### Knockdown of circADAMTS6 exhibits an anticancer effect on ESCC in vitro and vivo

**Figure 2:**
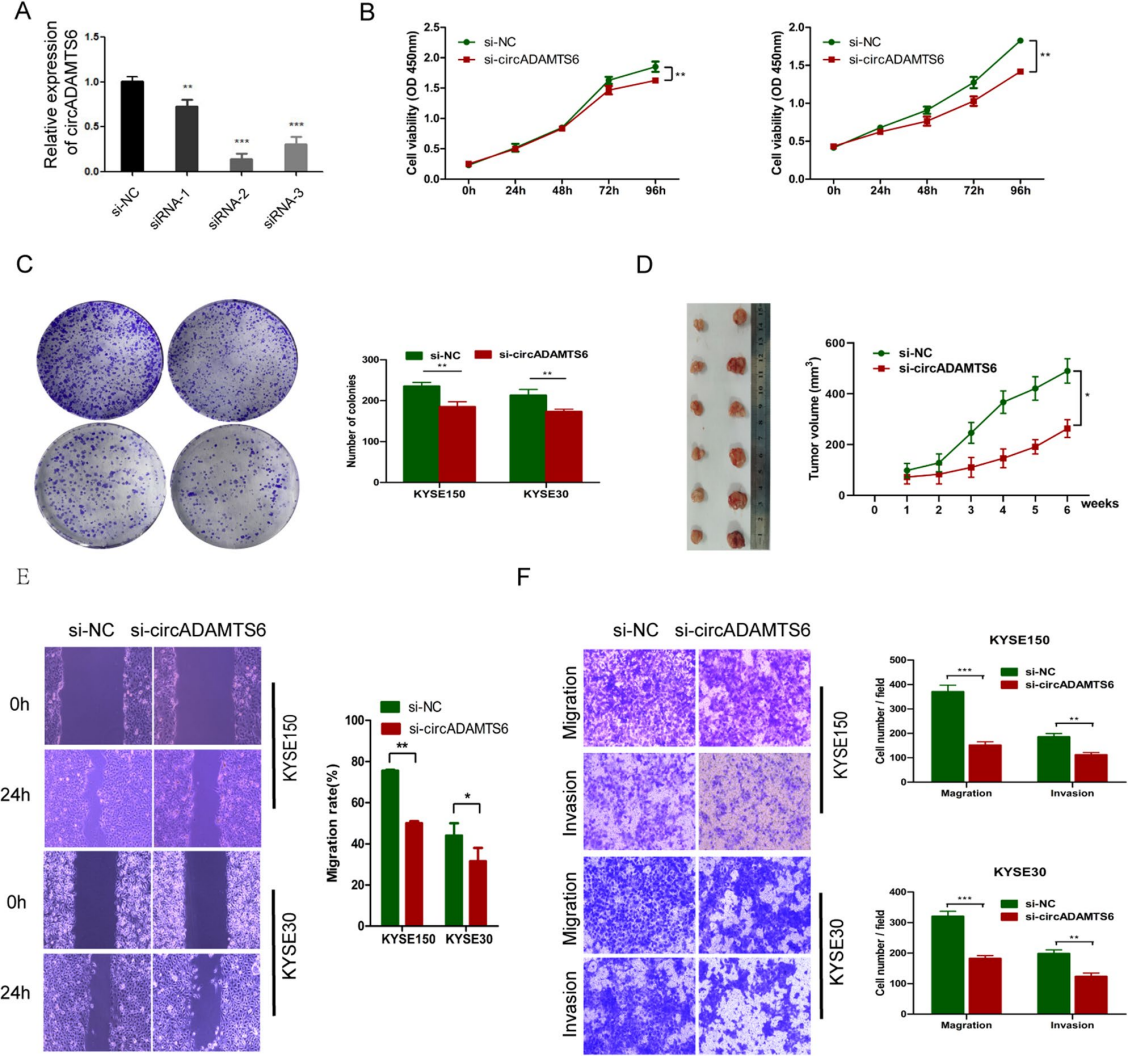
Knockdown of circADAMTS6 inhibits the ability of cell proliferation, migration and invasion in ESCC cells and tumor formation in vivo. (**A**) Relative expression level of circADAMTS6 transfected with si-NC and si-circADAMTS6 in KYSE150 cell. (**B**, **C**) Cell proliferation was assessed by CCK8 and colony formation assay after down-regulation of circADAMTS6. (**D**) Representative photographs and tumor growth curves of subcutaneous xenograft tumor model developed from KYSE150 cell with si-NC and si-circADAMTS6 (n = 6). The tumor volumes were calculated according to the formula (L × W^2^)/2. (**E**, **F**) Knockdown circADAMTS6 repressed migration and invasion of ESCC cells. (*P < 0.05, **P < 0.01, ***P < 0.001).

Initially, the siRNA that could specifically silence the back-splicing region of circADAMTS6 was constructed to assess the potential biological functions of circADAMTS6 in ESCC. Obviously, circADAMTS6 expression levels (Fig. 2A) were dramatically inhibited after transfection with circADAMTS6 siRNA (si-1, si-2 and si-3) compared with negative control siRNA (si-NC). We selected si-2 to explore the biological functions of circADAMTS6 in KYSE150 and KYSE30 cells. CCK-8 assay, colony formation assay, wound-healing assay and transwell assay were performed to evaluated the proliferation, migration and invasion of ESCC cells in vitro, and a nude mouse xenograft model was used to detect tumor formation in vivo. It showed that circADAMTS6 knockdown remarkably restrained the proliferation of ESCC cells in CCK8, colony formation assay (Fig. 2B,C) and xenograft nude mice model (Fig. 2D). Furthermore, results indicated that the migration and invasion capacity of ESCC cells were significantly blocked by wound-healing and transwell assay after transfection with si-2 (Fig. 2E,F). Together, these observations support that circADAMTS6 maybe a critical factor in the progression of ESCC.

### CircADAMTS6 is remarkably upregulated in ESCC tissues

**Figure 3:**
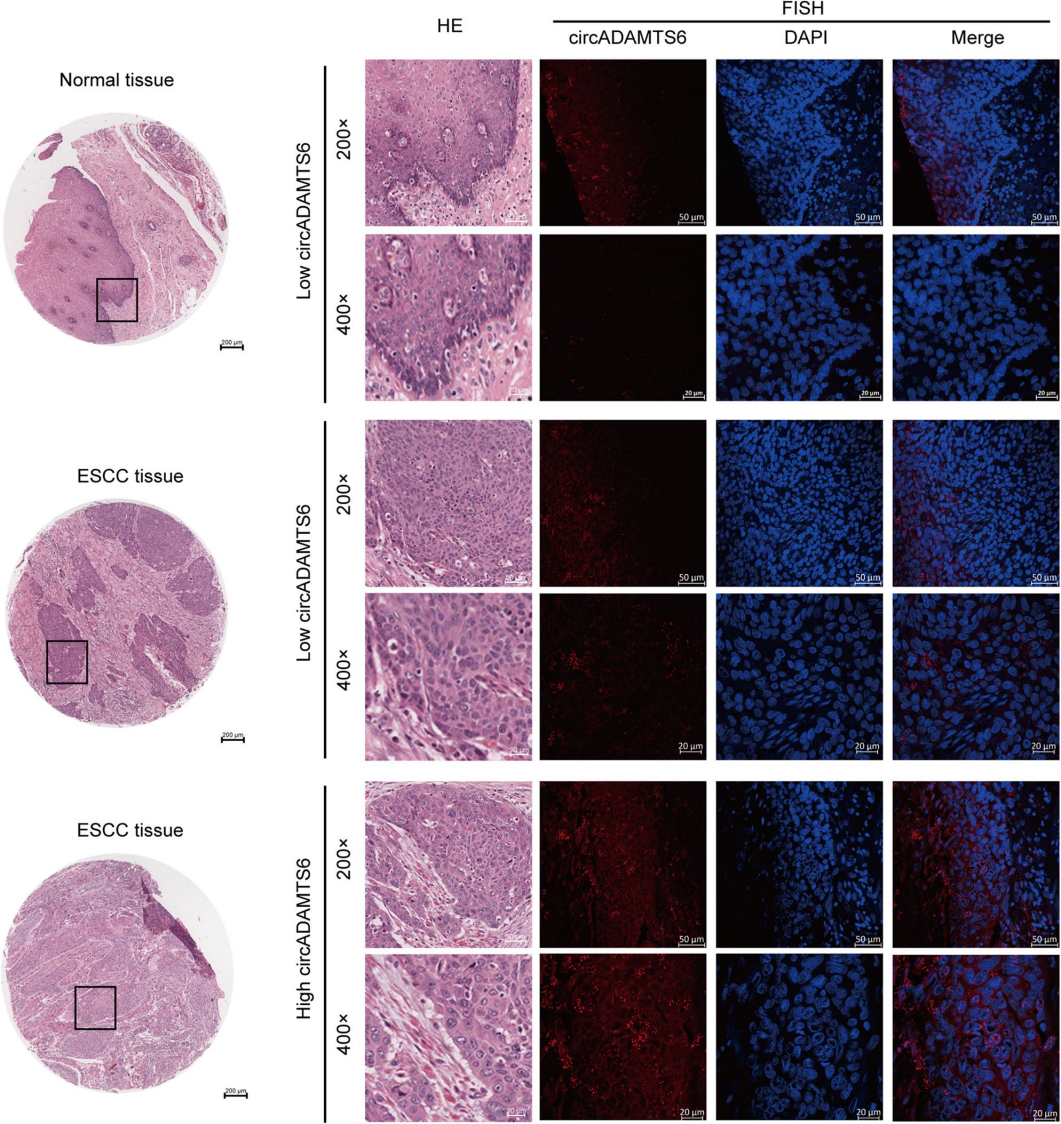
Different expression levels of circADAMTS6 in ESCC tissues and adjacent normal tissues. A comparison of two tissues, an increased expression of circADAMTS6 was discovered in ESCC tissues in our TMA-FISH results.

To analyze circADAMTS6 expression in ESCC tissues, fluorescence in situ hybridization (FISH) was applied by using the circADAMTS6 probe. 114 ESCC patients with clinicopathological and follow-up dates were obtained on Tissue Microarrays (TMAs). CircADAMTS6 positive cells were stained red and the nuclei were stained with DAPI in blue. Representative images of H&E and FISH staining of circADAMTS6 expression in ESCC tissues and adjacent normal tissues were displayed (Fig. 3). The outcomes indicated that circADAMTS6 was notably overexpressed in most ESCC tissues (n = 114; 81/114), which were great different from adjacent normal tissues (n = 66, 16/66).

### CircADAMTS6 is related to clinicopathological characteristics and OS in ESCC patients

**Figure 4:**
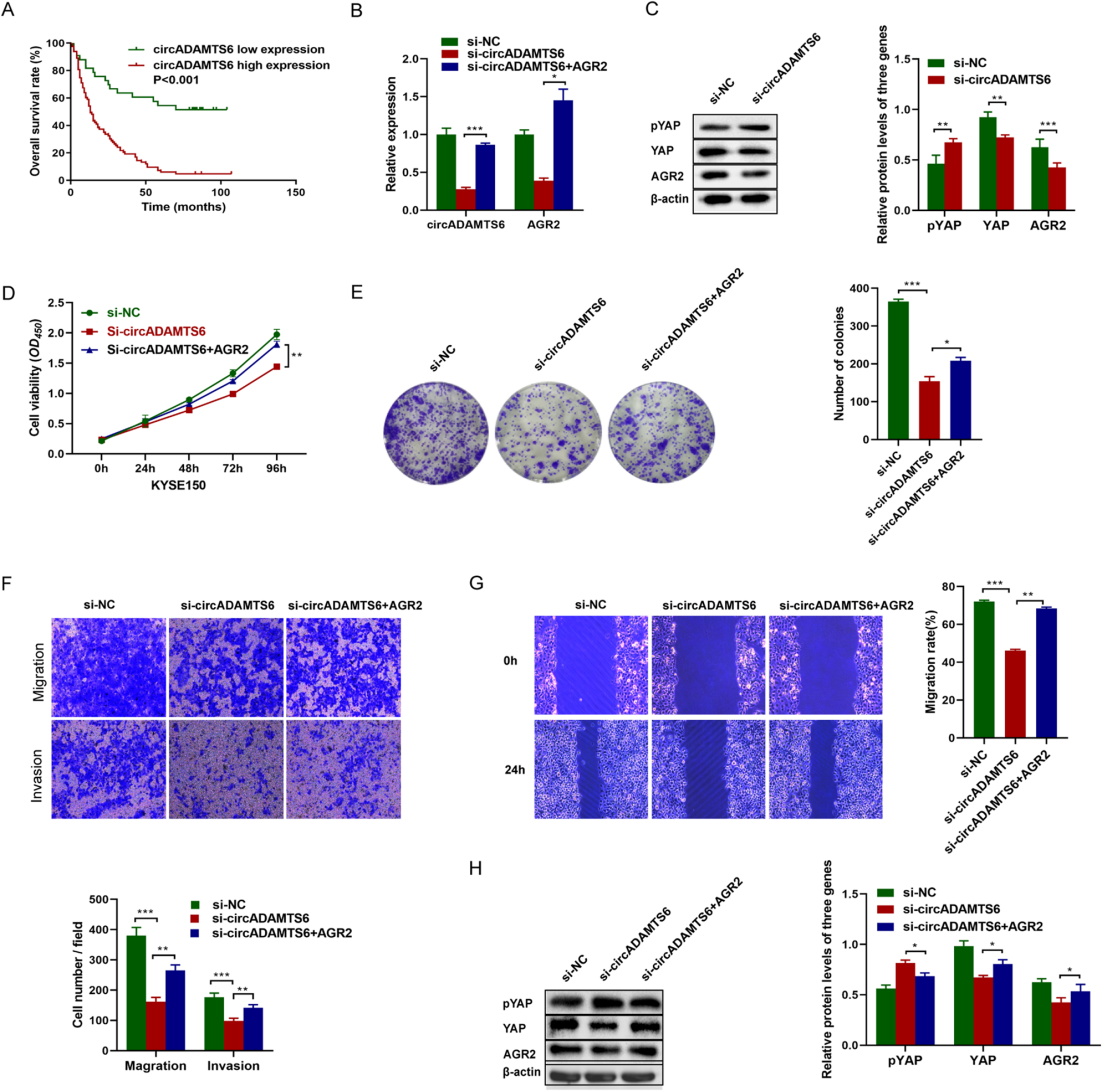
(**A**) Kaplan–Meier survival analysis indicated that circADAMTS6 high expression (*P* < 0.001) was significantly associated with shorter overall survival in ESCC cases. (**B**) CircADAMTS6 and AGR2 expression were measured after transfection of si-circADAMTS6 and co-transfection of AGR2 and circADAMTS6 by qRT-PCR. (**C**) Western blot analysis revealed that AGR2 and YAP proteins were significantly decreased after knockdown of circADAMTS6, while pYAP protein was significantly increased. (**D**–**G**) Rescue assays showed that transfection of AGR2 reversed the suppression of the proliferation (**D**, **E**), migration and invasion (**F**, **G**) caused by circADAMTS6 silencing in KYSE150 cell. (**H**) Western blot validated that the effect of pYAP, YAP and AGR2 proteins caused by si-circADAMTS6 could be rescued by transfection of AGR2.

The clinicopathological characteristics of ESCC patients in tissue microarrays were displayed in Supplementary Table 1. Our research discovered the positive expression rate of circADAMTS6 in ESCC tissues and adjacent normal tissues were 71.1% (81/114) and 24.2% (16/66), indicating that circADAMTS6 was more frequently expressed in ESCC tissues. The connection between circADAMTS6 levels and the clinicopathological characteristics of ESCC was investigated (Table 1). The high expression level of circADAMTS6 was closely related to advanced clinical stage, poor pathological grade, large tumor size and lymph node metastasis (*P* < 0.05), but not corrected with gender or age in ESCC tissues (*P* > 0.05). Multivariate analysis indicated that patients with higher circADAMTS6 expression had shorter overall survival time than those with lower circADAMTS6 expression, suggesting that circADAMTS6 was an independent prognostic factor in ESCC (Fig. 4A). These results provide a theoretical basis for further promoting circADAMTS6 as prognostic markers of ESCC.

**Table 1:** Univariate and multivariable analyses of prognostic factors in ESCC for overall survival.

Variable	Univariate analysis			Multivariate analysis		
	HR	*P* value	95% CI	HR	*P* value	95% CI
Expression of circADAMTS6 High versus Low	4.074	< 0.001	2.334–7.111	2.077	0.037	1.046–4.121
**Gender**						
Male versus Female	1.143	0.565	0.726–1.799			
**Age(years)**						
< 60 vs ≥ 60	2.177	0.003	1.295–3.658			
**Pathological grade**						
I versus II and III	3.249	< 0.001	2.067–5.107	1.650	0.068	0.964–2.824
**Tumor size (cm)**						
≤ 5 versus > 5	2.811	< 0.001	1.829–4.321	1.671	0.023	1.073–2.601
**Lymph node metastasis**						
No versus Yes	3.859	< 0.001	2.437–6.109	2.128	0.026	1.046–4.121
**Clinical stage**						
I and II vs III	3.761	< 0.001	2.320–6.097

### CircADAMTS6 promotes the progression of ESCC by regulating AGR2 and activating the expression level of the Hippo signaling pathway co-activator YAP

To explore whether AGR2 is regulated by circADAMTS6, the expression of circADAMTS6 and AGR2 were detected by si-circADAMTS6 in KYSE150 cell. qRT-PCR results showed that after transfection of si-circADAMTS6, the expression of AGR2 mRNA was distinguishedly lower than that in the control group (*P* < 0.05) (Fig. 4B). Consistently, Western blot analysis revealed that AGR2 and YAP proteins were significantly decreased after knockdown of circADAMTS6 (*P* < 0.05), while phosphorylated Yes-associated protein (pYAP) was significantly increased (*P* < 0.05) (Fig. 4C).

We further explored the relationship between AGR2 and circADAMTS6 by detecting whether AGR2 overexpressed could rescue the effect of knockdown circADAMTS6 in KYSE150 cell. Transfection of AGR2 overexpression plasmid significantly increased the expression level of AGR2 in KYSE150 cell (Fig. S5). For rescue experiments, AGR2 overexpression recovered the effects of circADAMTS6 knockdown on proliferation (Fig. 4D,E), migration and invasion(Fig. 4F,G) of KYSE150 cell . Besides, we measured the protein levels of YAP, pYAP and AGR2 using Western blotting (Fig. 4H). The results indicated that circADAMTS6 knockdown significantly decreased YAP and AGR2 levels and increased pYAP level. As expected, co-transfection of circADAMTS6 and AGR2 neutralized the effects of knockdown circADAMTS6 on YAP, pYAP and AGR2 expression.

The above results demonstrated that circADAMTS6 positively regulates AGR2 expression to promotes the progression of ESCC by activating the expression level of the Hippo signaling pathway coactivator YAP.

## Discussion

Increasing evidence indicates that circRNAs play vital roles in various physiological and pathological processes, suggesting that they may serve as potential diagnostic and predictive biomarkers in numerous diseases, including ESCC. CircRNAs interact with tumor-related miRNAs, proteases or signaling pathways, play important regulatory effects in the occurrence and development process of multiple tumors^20–22^. Traditional tumor markers are not sufficiently sensitive and effective for early ESCC detection, largely ESCC patients are diagnosed in the advanced stage and the overall 5-year survival rate is relatively poor^23–25^. Despite the characteristics and functions of many circRNAs have been studied in depth in recent years, the mechanism of circADAMTS6 in ESCC metastasis and prognosis remains unknown. Our research aim to investigate the potential role of novel circADAMTS6 in the development of ESCC and elucidate its underlying mechanism.

As a novel circRNA, circADAMTS6 was firstly reported to inhibit apoptosis in human chondrocyte by sponging miR-431-5p^26^. Subsequently, circADAMTS6 participates in IL-1β-induced human chondrocyte dysfunction though competing miR-324-5p and PI3K/AKT/mTOR signaling pathway^27^. In the study, we identified circADAMTS6 was notably high expressed in ESCC cells and tissues for the first time. In terms of its function and mechanism, circADAMTS6 promoted migration, proliferation and invasion of ESCC cells though the AGR2-YAP axis. Besides, high expression of circADAMTS6 was closely associated with pathological grade, tumor size, lymph node metastasis and poor prognosis. Correspondingly, silencing of circADAMTS6 was confirmed to suppress tumor growth in our xenograft mouse models. Notably, the above results demonstrated that the vital role of circADAMTS6, which may provide a potential therapeutic target for ESCC. As a widely used prognostic marker, the TNM classification is also the most universal tumor staging system in the world^28,29^. Then we guessed that the simultaneous use of TNM classification and molecular markers may predict the prognosis of ESCC patients more accurately. This is consistent with the research results of some scholars^30^.

As an evolutionarily conserved pathway, the Hippo/YAP signal transduction plays a vital role in organ size by regulating cell proliferation, apoptosis and metastasis in various diseases. Non-phosphorylation of YAP mediates the transcriptional function of cells, while phosphorylated YAP is retained in cytoplasm and cannot perform transcriptional functions. Furthermore, overexpression and nuclear localization of YAP has been detected in ESCC, lung cancer and breast cancer, and associated with a poor prognosis. AGR2 is an oncogene and involved in cell proliferation, invasion and tumour progression via microRNA, circRNAs and several pathways. It targets and regulates YAP and amphiregulin (AREG) to promotes tumor growth in lung adenocarcinoma^31^. Recently, AGR2 was described to promote tumor metastasis via activation of the mTOR/AKT signaling pathway^32^. Notably, high AGR2 expression levels was found in esophageal squamous tissue and ESCC cell lines, and was correlated with a worse prognosis in ESCC patients^33^. The above indicate the direction for further study of the mechanism of circRNAs in ESCC.

In conclusion, circADAMTS6 may play a vital role as an oncogene and serve as an independent molecular marker to promote early diagnosis and treatment of ESCC. However, article does not involve circADAMTS6 overexpression experiment due to the efficiency of circADAMTS6 formation is too low and the amplification fold of circADAMTS6 was much lower than that of the linear transcript. More works remain to be done for further explore to elucidate other mechanisms.

## Materials and methods

The Ethics Committee of the Fourth Affiliated Hospital, Hebei Medical University approved the study’s protocol and exempted informed consent (approval number: YB M-05–01). Our research was conducted in accordance with the Declaration of Helsinki. All animal experiments have been approved by the Animal Care and Use Committee of the Fourth Hospital of Hebei Medical University, and all experiments were carried out accordance with the guidelines. The study is reported in accordance with ARRIVE guidelines (www.arriveguidelines.org).

### Patients and clinical tissue samples

The ESCC tissue microarrays (TMAs: No. EsoS180Su08-XT18-031) were purchased from Shanghai Outdo Biotech Co., Ltd. (Shanghai, China), involving 114 cancer tissues and 66 adjacent normal tissues. None of the patients received any treatment before operation or diagnosed with other cancers. All patients underwent accurate surgery base on their clinical examinations combined with pathological diagnosis.

According to the hospital standard follow-up system, the patients were followed up and evaluated every 6 months after convalescence. July 31, 2015 was the deadline for follow-up evaluations. All the participants completed 0–107 months (average: 31.70 months) follow-up survey. The survival time was defined as the date of operation to the date of death or the deadline of follow-up.

The clinical features of the 114 patients were obtained from their medical records, such as age, gender, histological type tumor, pathological grade, primary tumor size and lymph node metastasis. According to the seventh edition of the American Joint Committee on Cancer (AJCC), metastasis status was assessed by the pathologic stage of the disease.

### Cell cultures and transfection

Esophageal cancer cell lines (KYSE150, KYSE30, KYSE170 and TE1) were provided by the Scientific Research Center of the Fourth Hospital of Hebei Medical University (Hebei, China), and cultured in RPMI1640 (GIBCO, USA) supplemented with 10% fetal bovine serum (GIBCO, USA), 100U/ml penicillin and 100 µg/ml streptomycin at 37 °C in humidified air containing 5% carbon dioxide.

KYSE150 and KYSE30 cells were maintained in six-well plates to 70 ~ 80% confluence, and transfected with circADAMTS6-siRNA (Geneseed Biotech Co, Guangzhou, China) (CTAGGTTAAAAAATGGCCA) or si-NC (negative control siRNA) using Lipofectmine 2000 (Invitrogen, USA). Measured the expression of circADAMTS6 by real-time RT-PCR at 48 h after transfection.

### DNA and RNA extraction, RNA RNase R treatment

Genome DNA was extracted from ESCC cells by using a simplified Proteinase K (Merck, Germany) digestion method. According to the manufacturer’s instructions, total RNA from cells was extracted using TRIzol Reagent (Invitrogen, USA) after transfection. RNase R treatment was managed using RNase R 3μ/mg for 15 min at 37 °C.

### Nucleic acid electrophoresis and quantitative real-time PCR

The cDNA was served as a template for Nucleic acid electrophoresis and quantitative real-time RT-PCR. All the primer sequences in our experiment were displayed in Supplementary Table 3. The small nuclear U6 and glyceraldehyde-3-phosphate dehydrogenase (GAPDH) were used as internal controls for circular RNA and linear RNA, respectively. The manufacturer’s instructions indicated that cDNA was synthesized from total RNA using GoScriptTM Reverse Transcription System (Promega, USA). Quantitative real-time PCR was performed performed with GoTaq® qPCR Master Mix (Promega, USA) using ABI QuantStudioTM 6 Flex. PCR products were separated on 2% agarose gels and imaged by UV irradiation (Fig. 1B). Besides, 2^−∆∆CT^ method was applied to compute the fold changes of the target gene expression.

### RNA fluorescence in situ hybridization (FISH)

RNA FISH was applied to explore circADAMTS6 expression in 114 ESCC tissues and adjacent normal tissues. Cy5-labelled circADAMTS6 probe (5’ biotin -ATGGCCATTTTTTAACCTAGTA- 3’ biotin) was designed and synthesized directly by Guangzhou Geneseed Biotech Co., Ltd. The FISH experiment was performed according to the manufacturer’s instructions. Images were obtained by using a fluorescence microscope (Carl Zeiss, Oberkochen, Germany) at room temperature.

### Wound healing assay

Diluted the cells to a density of 3 × 10^5^ cells per well and seeded in 6-well plates, incubated 48 h. After overnight incubation, cell mono-layer was scratched with a 20μL pipette tip and washed 3 times with PBS. Photos of the wound were taken at 0, 24 h under the microscope after scratched.

### Cell proliferation assay

To evaluated the proliferation of KYSE150 and KYSE30 cell lines by using CCK-8. Cells (5 × 10^3^ cells per well) in the logarithmic growth phase were seeded in 96-well plates and incubated at 37 °C. After 0 h, 24 h, 48 h, 72 h and 96 h, 10 μl CCK8 (Dojindo, Japan) was added to each well and incubated for 1 h. Finally, the absorption value of the whole wells was directly detected at 450 nm.

### Colony formation assay

Diluted the cells to a density of 1 × 10^3^ cells per well and seeded them into a 6-well plates and incubated at 37 °C, 5% CO_2_. After 7 days, cell colonies were stained with Giemsa and observed under the microscope.

### Transwell migration and matrigel invasion assay

To detect the capacity of migration and invasion of ESCC cells through using transwell chamber precoated with or without matrigel. Briefly, 5 × 10^4^ cells in the upper chamber were supplemented with serum-free RPMI1640 medium, whereas the lower chamber were cultivated in medium containing 10% FBS. Following overnight incubation, the migration cells and invaded cells were stained with Giemsa dye. The numbers of stained cells were calculated by a microscope (Leica, Germany) in five randomly selected fields.

### Animal in vivo assays

Four-week-old female BALB/c nude mice were purchased from SPF Biotechnology Co., Ltd (Beijing, China), and randomly divided into two groups (6 mice per group). To evaluate the function of circADAMTS6 in vivo, xenograft nude mice model was established by subcutaneously injecting KYSE150 cells (5 × 10^6^) into the right flank of mice. After tumor formation, 5 nmol si-RNA or si-NC was intratumorally injected into the two groups twice per week for three weeks. Tumor size was monitored weekly and volume was analyzed according to the formula:

$${\text{volume}} = \left( {{\text{With}}^{{2}} \times {\text{Length}}} \right)/{2}.$$

Six weeks later, all mice were sacrificed and tumor were harvested.

### Statistical analysis

Calculations in the study were carried out using SPSS Statistics 22.0 software (SPSS Inc, Chicago, IL, USA). Chi-square test was employed to assess the relevance between circADAMTS6 expression levels and the clinicopathological features of ESCC patients. Analyzed the survival outcomes relied on the Kaplan–Meier method and log-rank test, and evaluate the potential prognostic factors of overall survival based upon the Cox proportional hazards regression model. The results were expressed as the mean ± S.D., and analyzed by using Student's t-test. All statistical tests were two-sided, and dates in statistically significant were defined as *P* < 0.05.

## Supplementary Information

Supplementary Information.

## Data Availability

All data, models, and code generated or used during the study appear in the submitted article.
