# Supplementary material for: The circRNA circADAMTS6 promotes progression of ESCC and correlates with prognosis
Source: Sci Rep. 2022 Aug 12;12:13757. doi: 10.1038/s41598-022-17450-2 (PMC9374704; doi:10.1038/s41598-022-17450-2)
Supplement: Supplementary file 1 — Supplementary Information. [file 41598_2022_17450_MOESM1_ESM.pdf]

The circRNA circADAMTS6 promotes progression of ESCC and correlates with prognosis

Jing Bu, Lina Gu, Xin Liu, Xixi Nan, Xiangmei Zhang, Lingjiao Meng, Yang Zheng, Fei Liu, Jiali Li, Ziyi Li, Meixiang Sang, Baoen Shan

Correspondence to:
Prof. Baoen Shan
Department of Research Center
The Fourth Hospital of Hebei Medical University
Jiankang road 12
Email: baoenshan@hotmail.com

Keywords: ESCC, Expression, circADAMTS6, Prognosis

Supplemental Tables

Table 1 Correlations between the main clinicopathological features of ESCC patients after surgery and the expression of circADAMTS6.

Clinicopathological factors		CircADAMTS6 expression		X^2	P
		Low	High		
Gender				0.015	0.904
Men	83	24	58		
Woman	31	9	23		
Age/year				2.923	0.087
<60	29	12	17		
≥60	85	21	64		
Clinical stage				12.287	<0.001
I-II	44	21	23		
III	70	12	58		
Pathological grade				49.306	<0.001
I	50	31	19		
II-III	64	2	62		
Tumor size(cm)				6.896	0.009
≤5	61	24	37		
>5	53	9	44		
Lymph node metastasis				9.596	0.002
No	57	24	33		
Yes	57	9	48		

Table 2 Primer sequences used in this study

Gene		Primers
U6		F:5’- CTCGCTTCGCAGCACA -3’
		R:5’- AACGCTTCACGAATTGCGT -3’
GAPDH		F:5’-AGCCACATCGCTCAGACAC-3’
		R:5’-GCCCAATACGACCAAATCC-3’
circADAMTS6	Divergent	F:5’- TCCATTCTCTCCCACCAAAG -3’
		R:5’- CATAAGCAAAGCATTAGGCTGA -3’
	Convergent	F:5’- CCACAGAGGATTCCAAGCAT -3’
		R:5’- GTCATTCAGCCACCAAGGTT -3’

F: Forward primer; R: Reverse primer.

Note: Plasmid sequences of circADAMTS siRNA：CTAGGTTAAAAAATGGCCA

Table 3 The sequence of the AGR2 overexpression plasmid

**Gene**	**sequence**
**AGR2**	atggagaaaattccagtgtcagcattcttgctccttgtggccctctcctacactctggccagag<br>ataccacagtcaaacctggagccaaaaaggacacaaaggactctcgacccaaactgcccc<br>agaccctctccagaggttggggtgaccaactcatctggactcagacatatgaagaagctctat<br>ataaatccaagacaagcaacaaaccccttgatgattattcatcacttggatgagtgcccacaca<br>gtcaagctttaaagaaagtgtttgctgaaaataaagaaatccagaaattggcagagcagtttgt<br>cctcctcaatctggtttatgaaacaactgacaaacacctttctcctgatggccagtatgtcccca<br>ggattatgtttgttgacccatctctgacagttagagccgatatcactggaagatattcaaatcgt<br>ctctatgcttacgaacctgcagatacagctctgttgcttgacaacatgaagaaagctctcaagt<br>tgctgaagactgaattgtaa

Supplemental Figures

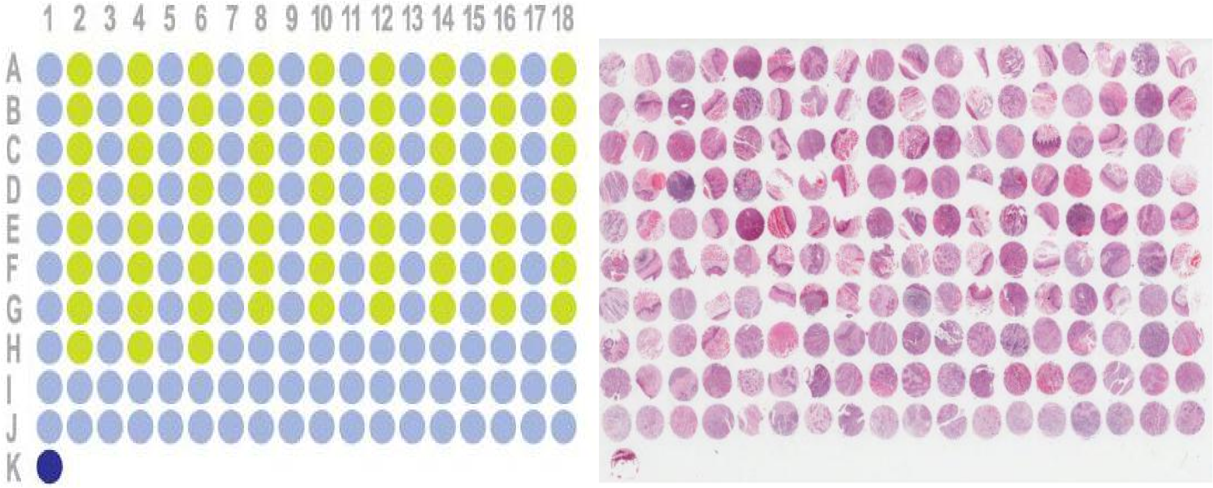

Figure 1. Schematic diagram (left) and HE-stained (right) of Tissue Microarrays (TMAs) (Blue represent ESCC tissues and green represent normal tissues).

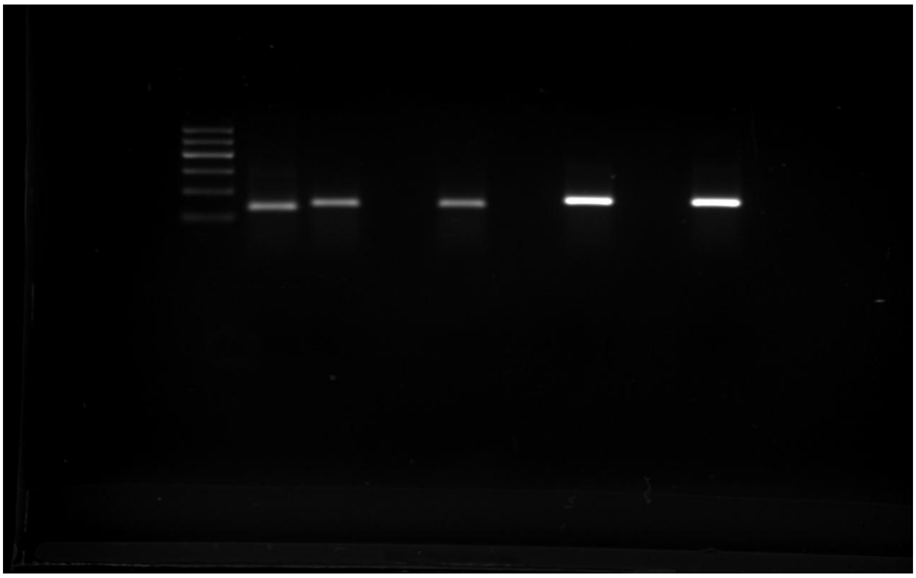

Figure 2. Full gel imaging picture of circADAMTS6 in KYSE150 cell and ESCC tissue.

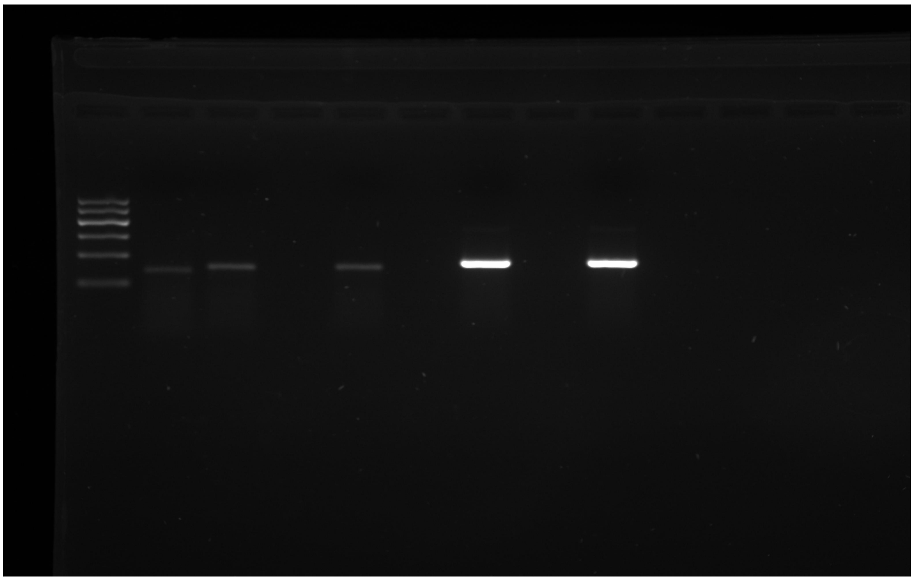

Figure 3. Full gel imaging picture of circADAMTS6 in KYSE150 cell.

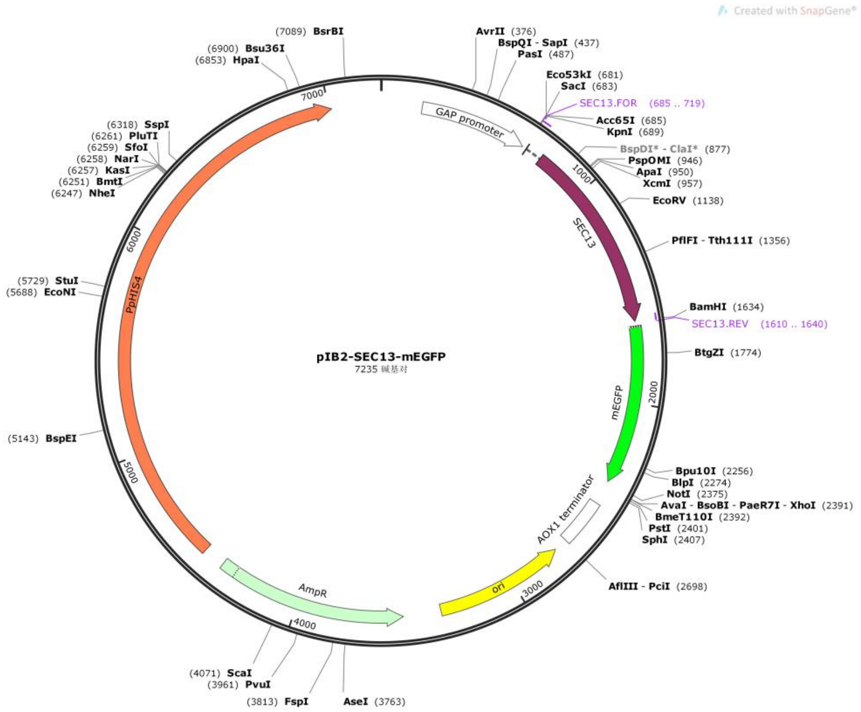

Figure 4. Schematic diagram of the construction of the AGR2 overexpression plasmid

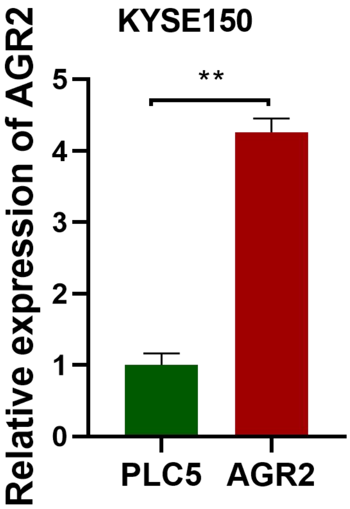

Figure 5. qRT-PCR was used to detect the transfection efficiency after overexpress of AGR2 in KYSE150 cell. (**$P<0.01$)
